# Identification and validation of a lenvatinib resistance-related prognostic signature in HCC, in which PFKFB4 contributes to tumor progression and lenvatinib resistance

**DOI:** 10.1186/s12876-025-03861-8

**Published:** 2025-04-23

**Authors:** Jinfeng Wang, Jianfei Shi, Lili Mi, Ning Li, Xin Han, Man Zhao, Xiaoling Duan, Guangjie Han, Jiaojiao Hou, Fei Yin

**Affiliations:** Department of Gastroenterology, The Fourth Hospital of Hebei Medical University, Tianshan Street 169, Shijiazhuang, Hebei China

**Keywords:** Hepatocellular carcinoma, Lenvatinib resistance-related genes, Prognostic signature, Tumor microenvironment, Treatment response

## Abstract

**Background:**

Drug resistance reflects the evolution of tumors and represents the leading factor behind recurrence and death. Lenvatinib is the first-line therapy for hepatocellular carcinoma (HCC), but its effectiveness is limited by rapid development of resistance. Therefore, we aimed to identify lenvatinib resistance-related genes and assess their influence on prognosis and treatment response in HCC.

**Methods:**

The GSE186191 dataset served as the discovery cohort to identify lenvatinib resistance-related genes. A Venn diagram analysis delineated the intersection between lenvatinib resistance-related genes and prognostic-associated genes derived from The Cancer Genome Atlas (TCGA) database. The LASSO Cox regression model was implemented to construct a multigene signature in the TCGA cohort. A nomogram was built by integrating the TNM stage and our prognostic model. The gene signature and nomogram were further validated using HCC patients from the International Cancer Genome Consortium (ICGC) cohort. Mutation signatures, therapeutic response, functional enrichment, and immune profile analyses were performed in the two groups. Two lenvatinib-resistant (LR) HCC cells were established using a concentration gradient increment method. PFKFB4 expression was detected via qRT-PCR and western blot assay. The CCK-8 assay and flow cytometry were utilized to evaluate the proliferation and apoptosis of LR cells under different interventions.

**Results:**

We developed a lenvatinib resistance-related gene signature (ALPK3, SLC2A2, CTSV, and PFKFB4), and demonstrated that’s a precise, independent, and specific prognostic model for HCC patients. High-risk patients were characterized by a predisposition to TP53 mutations, aggressive tumor features, and treatment resistance. The risk score was significantly associated with immune cell infiltration, immune checkpoint expression, angiogenesis, and tumor stemness. PFKFB4 was overexpressed in LR cells, and its knockdown significantly enhances the antiproliferative and pro-apoptotic effects of lenvatinib on resistant cells.

**Conclusions:**

The lenvatinib resistance-related prognostic signature exhibits strong predictive power for prognosis in HCC patients and may serve as an effective tool for guiding treatment decisions. PFKFB4 promotes tumor progression and lenvatinib resistance, highlighting its potential as a novel therapeutic target for HCC.

**Clinical trial number:**

Not applicable.

**Supplementary Information:**

The online version contains supplementary material available at 10.1186/s12876-025-03861-8.

## Background

Liver cancer is one of the most common visceral neoplasms and is ranked as the third cause of cancer-related death worldwide [1]. Hepatocellular carcinoma (HCC) represents the predominant pathological type of liver cancer and accounts for 75–80% of hepatic cancers [2]. Due to its insidious onset and strong invasiveness, HCC is characterized by delayed diagnosis and the development of resistant disease. Approximately 70% of HCC patients lose the opportunity for radical surgery, and despite surgical interventions, the recurrence rate within 5 years may be as high as 80% [3]. Systemic therapies have become the cornerstone of treatment for advanced-stage HCC, and estimates suggest that approximately 50–60% of patients diagnosed with HCC will ultimately receive systemic treatments [4]. However, intrinsic or acquired resistance may diminish the efficacy of systemic therapies, resulting in rapid tumor progression, recurrence, and ultimately an unfavorable prognosis [5]. As a complicated process, the dysregulated expression of genes and aberrant activation of signaling pathways play a pivotal role in the development of drug resistance and tumor advancement. Therefore, identifying resistance-related genes and assessing their effects on prognosis and drug sensitivity are vital steps in discovering novel targets to overcome drug resistance and improve the prognosis of HCC patients.

Lenvatinib is a representative multi-target tyrosine kinase inhibitors (TKIs) that primarily targets VEGFR 1–3, fibroblast growth factor receptor 1–4, PDGFR, RET, and KIT [6]. Whether administered alone or in combination with immunotherapy and local treatments, lenvatinib has demonstrated notable effectiveness in managing HCC [7, 8]. Nevertheless, most HCC patients inevitably develop drug resistance after approximately eight months of first-line lenvatinib treatment, ultimately leading to recurrence and metastasis [9]. Although extensive research have explored the mechanisms underlying lenvatinib resistance in HCC, including alterations in the tumor microenvironment(TME), cell apoptosis, cytokine overproduction, N6-threonylcarbamoyladenosine modification, and other factors [10], aberrant gene expression remains a fundamental contributor to drug resistance. However, few researchers have comprehensively investigated the role of lenvatinib resistance-related differentially expressed genes (LRRDEGs) in predicting the prognosis and treatment response of HCC patients.

In the present study, we acquired lenvatinib-resistant and -sensitive data from online databases and identified LRRDEGs. Lenvatinib resistance-related prognostic genes were subsequently screened by integrating LRRDEGs with HCC prognosis-associated genes from TCGA database. Based on these findings, we developed and validated the prognostic model and corresponding nomogram for HCC. Additionally, we performed a comprehensive comparison of clinical features, genetic mutation landscape, therapeutic response, tumor microenvironment, stemness score, and functional enrichment analysis in high-risk and low-risk groups. The results revealed significant distinctions between the two groups across all these aspects. Finally, we validated the prognostic significance of the identified genes using both internal and external datasets. Moreover, in vitro experiments confirmed the crucial role of PFKFB4 in lenvatinib resistance. Therefore, our findings provide a novel prognostic biomarker and predictive tool that can guide personalized treatment for HCC patients.

## Materials and methods

### Data acquisition and processing

The mRNA expression profiles of 371 HCC samples in the liver hepatocellular carcinoma (LIHC) cohort were downloaded from The Cancer Genome Atlas (TCGA) (https://portal.gdc.cancer.gov) database, and corresponding clinical information was obtained from cBioPortal (http://www.cbioportal.org). Patients without overall survival (OS) data were rigorously excluded, resulting in a refined training cohort of 365 patients for subsequent prognostic model development and statistical analysis. For external validation, the ICGC-LIRI-JP cohort, consisting of mRNA expression profiles and clinical information from 231 HCC patients, was obtained from the ICGC database (https://dcc.icgc.org/). The detail clinicopathological characteristics regarding age, gender, tumor grade, TNM stage, alpha-fetoprotein (AFP) level, and survival status of the TCGA and ICGC database are summarized in Table S1.The gene expression data of GSE186191, GSE25097, GSE57957, and GSE104580 was downloaded from the Genomics Expression Omnibus (GEO) database (https://www.ncbi.nlm.nih.gov/geo/). The mRNA sequencing data of the TCGA-LIHC and ICGC-LIRI-JP cohorts were converted into transcripts per kilobase million (TPM) values, and then log2(TPM + 1) transformed, which was recommended as the most accurate quantification method with minimal statistical biases [11]. The “Combat” function of the “sva” package was used to eliminate the batch effects between the two datasets.

### Screening of LRRDEGs

The dataset (GSE186191), including RNA-seq data for parental (Hep3B, Huh7) and lenvatinib-resistant HCC cells (Hep3B-LR, Huh7-LR), was retrieved from the GEO database. To obtain LRRDEGs, the R package “DEseq2” was used in the standard comparison model. The differentially expressed genes (DEGs) threshold was set at an absolute log2-fold change (FC) ≥ 1 and a false discovery rate (FDR) < 0.05.

### Identification of prognostic related genes in HCC patients

“DEseq2” R package was also used to obtain DEGs between HCC(*n* = 365) and normal tissues (*n* = 50) in the TCGA database. An FDR < 0.05 and|log2FC|>1 were criteria for statistical significance. Then the “survival” analysis package was used for univariate Cox regression analysis to screen differentially expressed genes that were related to prognosis.

### Establishment and validation of a lenvatinib resistance-related prognostic signature 

We identified the intersection between the LRRDEGs and prognostic-related genes in HCC patients using a Venn diagram. Then, a lenvatinib resistance-related prognostic signature was established using a LASSO-penalized Cox regression analysis. The lenvatinib resistance-related prognostic score (LRRPS) was calculated as follows: risk score = (exprgene1 × coefficientgene1) + (exprgene2 × coefficientgene2) + ···+ (exprgeneN × coefficientgeneN). The median risk score was utilized as the cut-off value to stratify patients with HCC into high-LRRPS and low-LRRPS groups, revealing a significant difference in prognosis between the two groups. Kaplan-Meier survival curves and time-dependent receptor operating characteristic (ROC) curves were performed to assess the predictive performance of the prognostic signature for overall survival. Additionally, principal component analysis (PCA) was conducted based on the expression profiles of genes within the signature using the “prcomp” function of the “stats” R package. To further validate the model, an independent dataset (ICGC-LIRI-JP), comprising 231 HCC patients, was utilized as an external validation cohort.

### Construction and evaluation of a predictive nomogram

Univariate and multivariate COX analyses were used to determine the independence of LRRPS in predicting the OS of HCC patients. Variables significantly associated with survival in the univariate analysis were incorporated into a multivariable Cox proportional hazards model. Furthermore, a predictive nomogram integrating LRRPS and clinical features was developed to estimate the survival risk of HCC patients. The calibration curve, generated using the “rms” R package, was employed to assess the predictive accuracy of the nomogram. Additionally, the time-dependent concordance index (C-index) was calculated to evaluate the sensitivity and specificity of the nomogram in predicting OS, compared to using a standalone predictor.

### Mutation landscape

The somatic mutation data of HCC patients in the TCGA cohort were retrieved from the GDC database. As described in previous literature [12], we processed and visualized the downloaded MAF files of simple nucleotide variation using the “maftools” package in the R environment. Additionally, the tumor mutation burden (TMB) and mutant-allele tumor heterogeneity (MATH) score for tumor samples in the TCGA-LIHC cohort were computed using the “maftools” package.

### Prediction of therapeutic response

To improve personalized treatment strategies, we utilized the R package “OncoPredict” to evaluate the chemotherapeutic response of HCC patients in the two different risk score groups. The data of cell lines in the Cancer Therapeutics Response Portal (CTRP) V2.1 were used as the training cohort. We also assessed the relationship between LRRPS and responsiveness to transcatheter arterial chemoembolization (TACE) using the GSE104580 dataset.

### Tumor microenvironment and stemness score analysis

The tumor microenvironment plays a pivotal role in both tumor progression and treatment response [13]. We estimated the relative proportions of various cell subsets in tissue using CIBERSORTx method. The stromal scores, immune cell scores, microenvironment scores were calculated using the ESTIMATE analysis. Based on RNA-seq datasets from TCGA, the one-class logistic regression (OCLR) algorithm [14] was applied to compute the mRNA-based stemness index (mRNAsi) of HCC patients. The association between the mRNAsi and the key lenvatinib resistance-related prognostic genes were assessed by the Spearman rank correlation test.

### Functional enrichment analysis

The LRRDEGs were uploaded to the Database for Annotation, Visualization, and Integrated Discovery (DAVID)( https://davidbioinformatics.nih.gov/) to analyze the Kyoto Encyclopedia of Genes and Genomes (KEGG) pathways and biological processes associated with lenvatinb resistance. Gene set enrichment analysis (GSEA) was used to explore the pathway activities in different risk-score groups. Gene expression data were loaded into GSEA, and c2.cp.kegg.v2023.1.Hs.symbols.gmt was selected as the gene set database. The pathways with the following criteria were regarded to be significantly enriched: nominal *p*-value < 0.05, FDR < 0.25, and normalized enrichment score > 1 and < -1.

### Cell lines and cell culture

The Huh7 and HepG2 cell lines with STR profiling, were obtained from Meisen Biotech, Zhejiang, and cultivated in Dulbecco’s modified Eagle’s medium (DMEM) supplemented with 10% foetal bovine serum at 37 °C and 5% CO2. The concentration gradient increment method [15] was employed to establish lenvatinib-resistant HCC cells. Specifically, Huh7 and HepG2 cells were exposed to lenvatinib (HY-10981, MCE) at initial concentrations of 0.2µM and 2µM, respectively. The concentration of lenvatinib was progressively increased, reaching 10µM and 25µM, respectively, in the two cell lines. Approximately 8 months later, we successfully established two lenvatinib-resistant HCC lines, named lenvatinib-resistant Huh7 cells (Huh7-LR) and lenvatinib-resistant HepG2 cells (HepG2-LR). The two resistant cell lines were consistently cultured in the presence of appropriate concentrations of lenvatinib to sustain their drug resistance.

### siRNA transfection

A PFKFB4 siRNA (siPFKFB4) was designed by GenePharma (Shanghai GenePharma Co., Ltd. China). When the cell density reaches 60–70%, they were transfected with siPFKFB4 or control siRNA (siNC) using TransIntro^®^ EL transfection reagent(TransGen Biotech, China)according to the instructions. Forty-eight hours after transfection, western blot analysis was performed to verify the knockdown efficiency. The sequence of siPFKFB4 was as follows: 5′- GGACUUCAUGAGGCGCAUUTT-3′.

### Western blotting analysis

The cells were collected and lysed using a RIPA Lysis Buffer (Solarbio Science & Technology Co., Ltd, Beijing, China). Equal amounts of protein from each sample were loaded on 8% or 10% sodium dodecyl-sulphate polyacrylamide gel electrophoresis and transferred onto an Immobilon^®^-P Transfer membrane (IPVH00010, Merck Millipore Lt). After blocking with 5% non-fat milk, the labelled membrane was incubated with PFKFB4 antibody (1:1000, Cat. No. GTX107755, GeneTex) at 4 °C overnight. The membranes were then incubated with HRP-conjugated secondary antibodies for 2 h at room temperature. Finally, the membranes were incubated with enhanced chemiluminescence reagent and the bands were detected. For the verification of PFKFB4 knockdown efficiency in HepG2-LR cells, membranes were cut prior to antibody incubation and the membranes were analyzed using Amersham Imager600 (GE, Boston, USA). Intact membranes were employed for all other western blot experiments and were analyzed using Tanon 4800 automated imaging system (Tanon Science & Technology Co., Ltd., Shanghai, China). GAPDH was used as an internal reference.

### Quantitative real-time polymerase chain reaction (qRT-PCR)

Total RNA was extracted from parental and lenvatinib-resistant cells using TRIzol reagent (Ambion, US) and reverse-transcribed to cDNA using the HiFiScript cDNA RT KIT (Proteinssci Biotech Co., Ltd, Shanghai, China). The qRT-PCR was performed by using the MagicSYBR Mixture with a CFX96 Touch™ real-time PCR detection system (Bio-Rad Laboratories, Hercules, CA, USA) following the manufacturer’s protocol. The primers used in this study were manufactured by Sangon Biotech (Shanghai, China) with the following sequences: PFKFB4-F: GGCTCCTGACCTGCTGCTAAG; PFKFB4-R: CTGGCGAGAGTGAACACCTAAGAG. ACTIN-F: TCGTGCGTGACATTAAGGAGAAGC; ACTIN -R: GGCGTACAGGTCTTTGCGGATG. The results were normalized to β-actin expression and are presented as relative mRNA expression levels. The relative expression of the targeted gene was determined by using the 2-△△CT comparative method.

### Cell viability assay

Cells were seeded into 96 well plates at a density of 3000 cells and then placed in the incubator for 24 h. Cells were cultured and subjected to different interventions for 24, 48, 72 and 96 h, and their proliferative viability was measured using Cell counting kit-8 (CCK8) assay. In addition, the half-maximal inhibitory concentration (IC50) of lenvatinib was determined in both parental and resistant cells using the CCK-8 assay. After overnight incubation to allow cells to adhere to the plate, cells were exposed to various concentrations of lenvatinib for 72 h. Subsequently, 10 µl of CCK-8 reagent was added to each well, shielded from light, and the plate was further incubated for 2 h. Finally, the optical density (OD) values were measured at a wavelength of 450 nm using an enzyme marker. Standard curves were generated using GraphPad Prism (V 8.0) software.

### Cell apoptosis assay

The cells were seeded at a density of 1 × 10^6^ cells per well in a 6-well plate and cultivated overnight. After cultured 72 h, the cells were collected and stained with 5 µl Annexin V-FITC and 10 µl propidium iodide for 20 min and then analyzed using flow cytometry (Beckman Coulter, USA).

### Clonogenicity assay

To compare the clonogenicity of parental and lenvatinib-resistant cell lines, 1 × 10^3^ cells per well were seeded in a 6-well plate and continuously exposed to culture medium containing lenvatinib for two weeks. After fixing in methyl alcohol for 30 min and staining with crystal violet for 20 min, the colonies were photographed using a camera and analyzed using Image J Software.

### Statistical analysis

Statistical analyses and visualizations were performed with SPSS 25.0 (IBM Corp), R software (version 4.2.1) and Prism 10.0 (GraphPad, San Diego, CA, USA). Unpaired Student t-tests or Mann-Whitney U tests (also called the Wilcoxon rank-sum test) are used to compare the differences between the two groups. One-way ANOVA or Kruskal-Wallis tests were used for comparisons of more than two groups. Best-fit normalized dose-response curves for lenvatinib were used to calculate IC50 values with 95% confidence intervals in GraphPad Prism. A *P* value < 0.05 was considered statistically significant.

## Results

### Identification of lenvatinib resistance-related prognostic genes

**Fig. 1:**
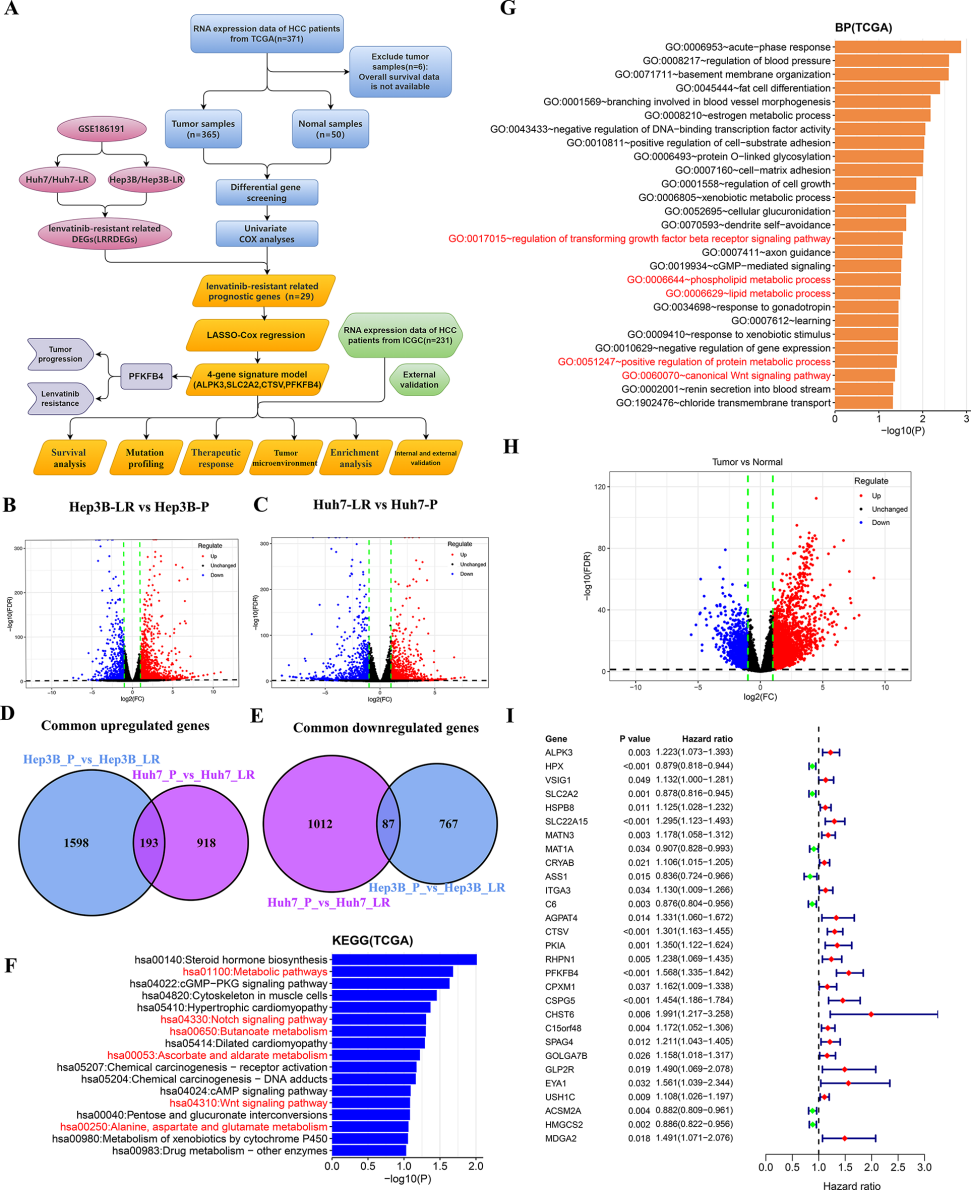
Identification of lenvatinib resistance-related prognostic genes. **A** Schematic overview of the workflow in this study. **B** Volcano plot of DEGs (Hep3B-LR vs. Hep3B-P). **C** Volcano plot of DEGs (Huh7-LR vs. Huh7-P). **D**, **E** Wayne diagram showing common upregulated DEGs (**D**) and common downregulated DEGs (**E**). **F**, **G** KEGG pathways(**F**) and biological processes(**G**) associated with LRRDEGs. **H** Volcano plot of DEGs (tumor vs. normal) in TCGA database. **I** Forest plots of 29 lenvatinib resistance-related prognostic genes

As shown in Fig. 1A, we designed this study to develop a prognostic signature related to lenvatinib resistance and investigate its potential value for predicting prognosis and treatment response in HCC. To identify genes potentially involved in lenvatinib resistance, we analyzed the publicly available dataset GSE186191 [16]. In this dataset, two lenvatinib-resistant human HCC cell lines (Huh7-LR and Hep3B-LR) were established by exposing parental HCC cells to incrementally increasing lenvatinib concentrations. We analyzed the DEGs in the mRNA expression profiles between parental and lenvatinib-resistant HCC cells (Fig. 1B-C). In total, 193 common upregulated (Fig. 1D) and 87 common downregulated (Fig. 1E) LRRDEGs were screened out with a threshold of FDR < 0.05 and|log2 FC| ≥ 1. Pathway enrichment analysis was conducted to gain insight into the function of LRRDEGs. KEGG pathway enrichment analysis revealed that the LRRDEGs were predominantly enriched in metabolism pathway, Notch signaling pathway and Wnt signaling pathway (Fig. 1F). Additionally, gene ontology analysis of biological processes demonstrated significant enrichment of LRRDEGs in terms related to regulation of transforming growth factor beta (TGF-β) receptor signaling pathway, metabolic processes and the canonical Wnt signaling pathway (Fig. 1G). These findings collectively suggest that the aforementioned signaling cascades and biological process may play pivotal roles in the development of lenvatinib resistance, potentially providing novel therapeutic targets for overcoming drug resistance in clinical settings. To obtain prognostic-related genes in patients with HCC, the 3641 DEGs (Fig. 1H) between normal liver tissue and HCC tissue were subjected to univariate COX regression analysis in the TCGA database. Subsequently, we intersected the prognostic-related genes in HCC with the LRRDEGs, identifying 29 lenvatinib resistance-related prognostic genes for further analysis (Fig. 1I).

### Construction and validation of a lenvatinib resistance-related prognostic signature

**Fig. 2:**
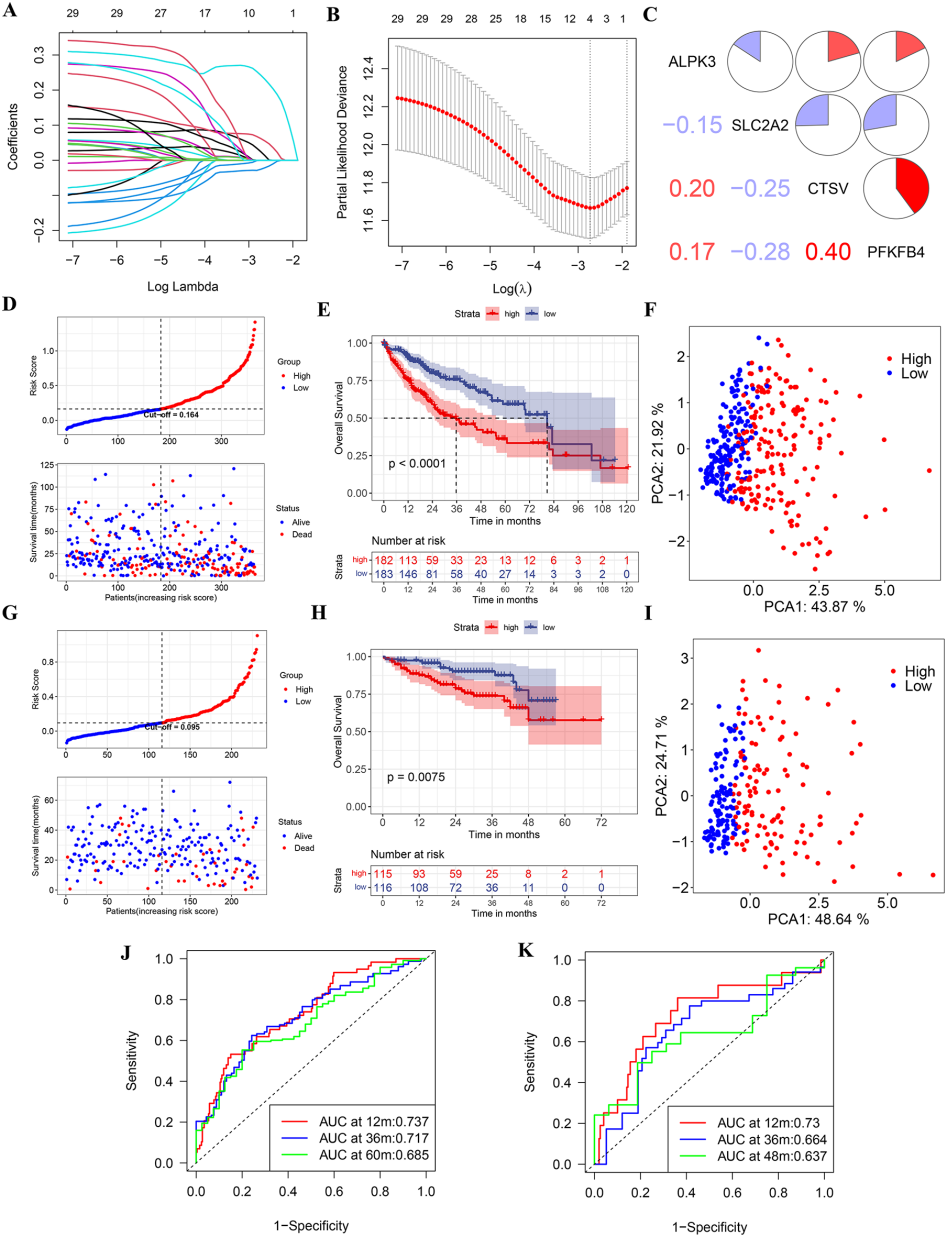
Construction and validation of LRRPS. **A** Coefficient values of genes. **B** The coefficient plot was plotted against the log(lambda) values. **C** A matrix correlation analysis of four selected genes. **D**, **G** Median risk score values and the correlations between risk score and OS in the TCGA (**D**) and ICGC (**G**) database. **E**, **H** Kaplan-Meier analysis of OS in the TCGA (**E**) and ICGC (**H**) database. **F**, **I** PCA in TCGA (**F**) and ICGC (**I**) database. **J**, **K** Time-dependent ROC curves and AUC analyses in the TCGA (**J**) and ICGC (**K**) database

Next, a 10-fold cross-validated Lasso-Cox regression was performed to construct a lenvatinib resistance-related prognostic signature using the expression profile of the 29 genes mentioned above (Fig. 2A-B). The LRRPS was calculated as follows: risk score = ALPK3*0.024 + SLC2A2*(-0.025) + CTSV*0.062 + PFKFB4*0.242. As depicted in Fig. 2C, the pairwise correlation coefficients among the four genes were all below 0.5, suggesting a weak correlation among the genes themselves. We then classified the patients into either a high-LRRPS group (*n* = 182) or a low-LRRPS group (*n* = 183) based on the median risk score. The scatter plot indicated that LRRPS were negatively associated with survival time (Fig. 2D). Furthermore, the Kaplan-Meier analysis showed that patients in the high-LRRPS group exhibited significantly poorer overall survival compared to those in the low-LRRPS group (Fig. 2E). The above results indicate that patients with high LRRPS have an increased risk of mortality compared to those with low LRRPS. Therefore, in subsequent analyses, we defined patients with low LRRPS as the “low-risk” group and those with high LRRPS as the “high-risk” group. The PCA analysis revealed distinct distributions of patients with different risk scores, further supporting the robustness of the classification (Fig. 2F). The predictive performance of the risk score for overall survival was assessed using time-dependent ROC curves, with the area under curve (AUC) achieving values of 0.737 at 1 year, 0.717 at 3 years, and 0.685 at 5 years (Fig. 2J). To validate the robustness of the model established using the TCGA cohort, patients from the ICGC cohort were stratified into high- or low-risk groups based on the median value calculated using the same formula as that of the TCGA cohort. Consistent with the results from TCGA database, patients in the high-risk group exhibited significantly poorer OS in the ICGC cohort (Fig. 2G-H). Likewise, PCA analysis confirmed that patients in two subgroups were distributed in discrete directions (Fig. 2I). Besides, the AUC of the 4-gene signature was 0.730 at 1 year, 0.664 at 3 years, and 0.637 at 4 years in ICGC database (Fig. 2K). The consistent findings across both the discovery (TCGA) and validation (ICGC) datasets suggest that the risk scores possessed exceptional specificity and sensitivity in predicting the prognosis of HCC patients.

### Construction and validation of the nomogram predicting OS for HCC patients

**Fig. 3:**
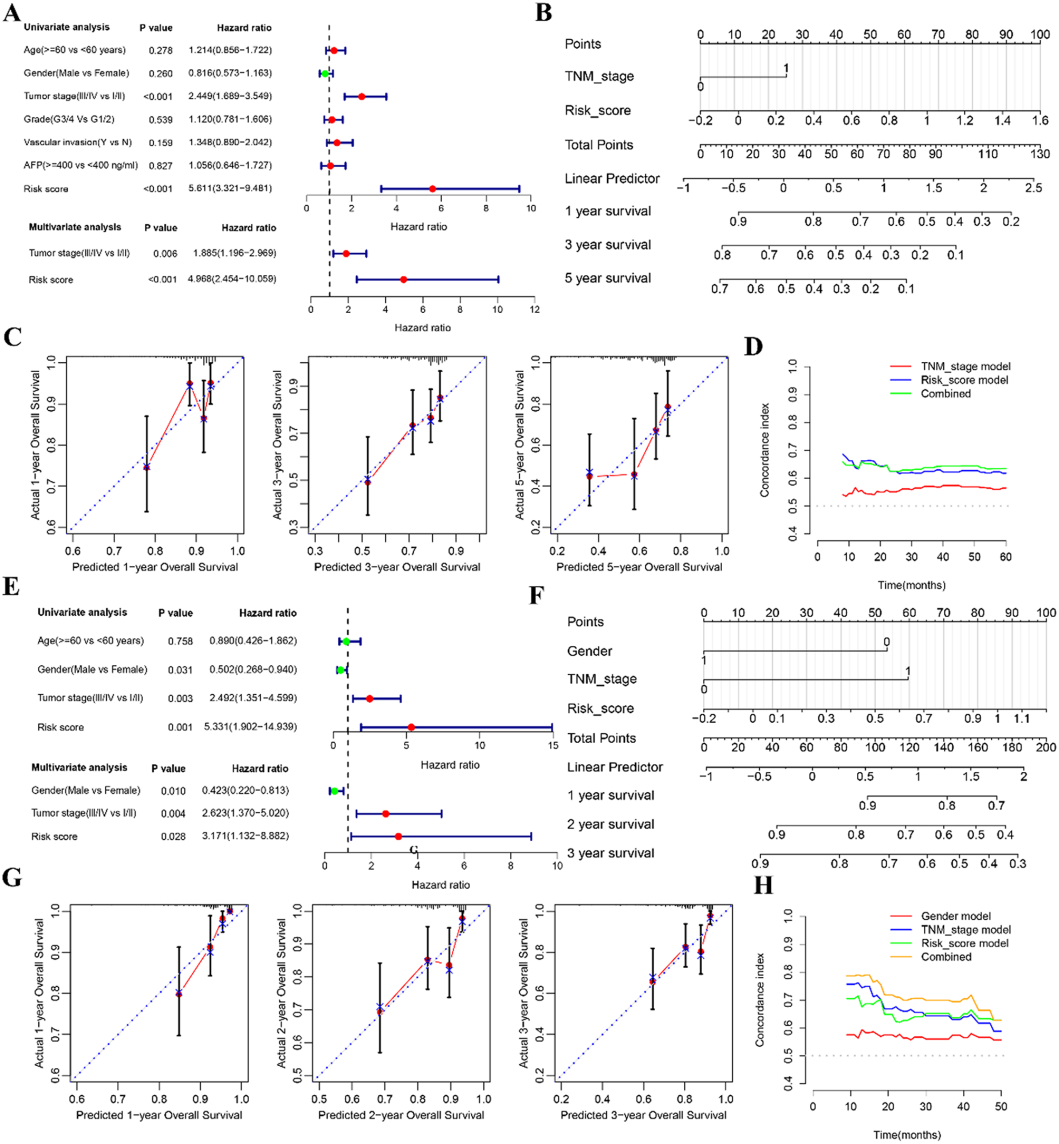
Construction and validation of the nomogram predicting OS for HCC patients. **A**, **E** Forest plot of the univariate and multivariate Cox regression analyses in TCGA (**A**) and ICGC (**E**) database. **B**, **F** The nomogram plot based on independent prognostic factors in TCGA (**B**) and ICGC (**F**) database. **C**, **G** Calibration curves of the nomogram for predicting survival rates in TCGA(**C**) and ICGC (**G**) databases. **D**, **H** The time-dependent C-index of prognostic model in TCGA (**D**) and ICGC (**H**) database

To validate the risk score as an independent prognostic biomarker for OS, COX proportional hazards regression analysis was conducted in both the TCGA and ICGC cohorts. As shown in Fig. 3A, univariate and multivariate analyses revealed that TNM stage (*P* = 0.006, HR = 1.885, 95% CI:1.196–2.969) and risk score (*P* < 0.001, HR = 4.968, 95%CI: 2.454–10.059) were correlated with OS of HCC patients, suggesting that the risk score was an independent prognostic indicator of HCC in the TCGA database. Similarly, the risk score (*P* = 0.028, HR = 3.171,95%CI:1.132–8.882) was also identified as an independent risk factor for OS in the ICGC database (Fig. 3E). Furthermore, we established a prognostic nomogram model based on the significant factors found in the above multivariate analysis (Fig. 3B, F). We used the calibration plot to evaluate the accuracy of the nomogram model, which showed a close alignment between the predicted and observed overall survival rates, indicating the model’s high predictive reliability (Fig. 3C and G). Additionally, the time-independent C-index illustrated the superiority of our nomogram model over individual clinical variables (Fig. 3D, H).

### The association between LRRPS and clinicopathological characteristics

**Fig. 4:**
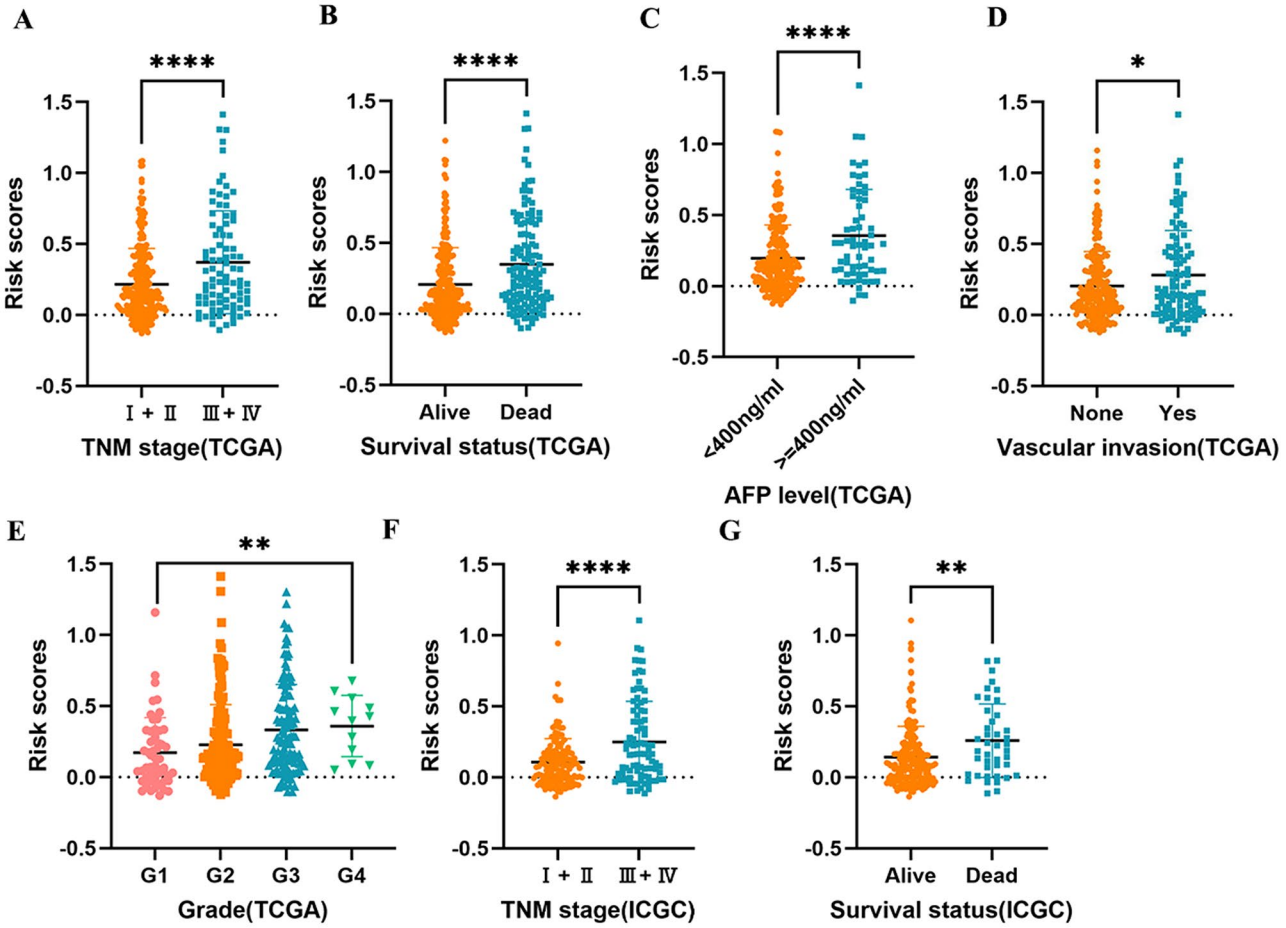
The association between LRRPS and clinicopathological indicators. **A-E** The association between LRRPS and TNM stage(**A**), survival status(**B**), AFP level(**C**), vascular invasion(**D**) and grade(**E**) in TCGA database. **F**, **G** The association between LRRPS and TNM stage(**F**) and survival status(**G**) in ICGC database. *P < 0.05, **P < 0.01, **** *P* < 0.0001

Subsequently, we investigated the correlation between clinicopathological features and LRRPS. Notably, patients with fatal outcomes and more aggressive tumor characteristics—including higher histological grade, markedly elevated AFP levels, presence of vascular invasion, and more advanced TNM staging—demonstrated significantly elevated LRRPS values in the TCGA cohort (Fig. 4A-E). Concordantly, in the ICGC database, patients with advanced TNM stages and fatal outcomes exhibited significantly higher risk scores, further validating this association (Fig. 4F,G).

### Gene mutation landscape in high-and low-risk groups

**Fig. 5:**
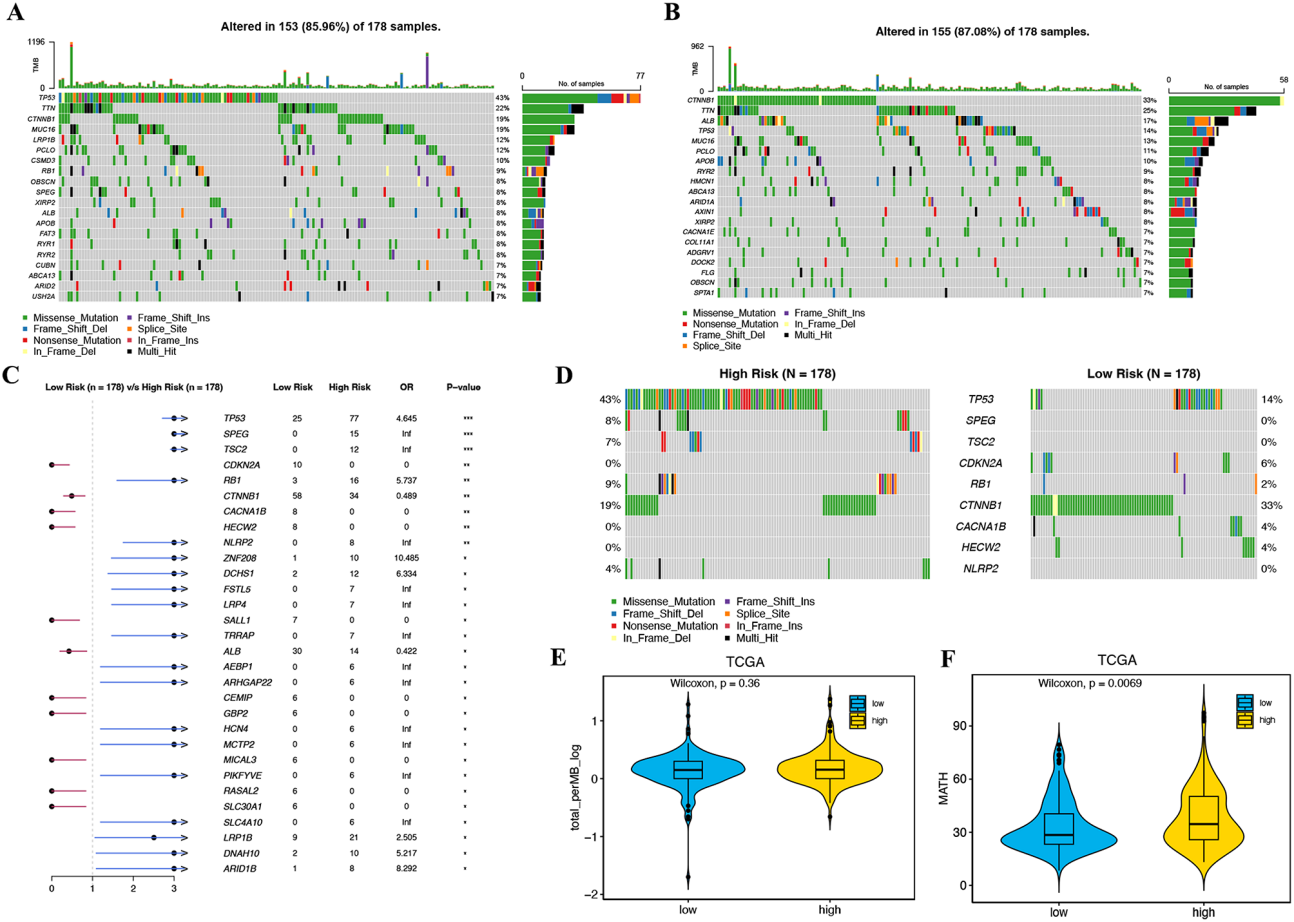
Gene mutation landscape in high- and low-risk groups. **A**, **B** Oncoplots of the mutated genes in the (**A**)high-risk and (**B**) low-risk subgroups. **C** Forest plot of the differentially mutated genes between the high- and low-risk groups. **D** Mutated genes with significant differences between high-risk and low-risk groups. **E**, **F** TMB (**E**) and MATH (**F**)scores in the high- and low-risk groups

To compare gene mutation profiles between the high-risk and low-risk groups, simple nucleotide variation data were downloaded from the GDC database and processed with the “maftools” package in R. As shown in Fig. 5A, the top five genes with the highest mutation frequencies in the high-risk group were TP53 (43%), TTN (22%), CTNNB1 (19%), MUC16 (19%), and LRP1B (12%), while in the low-risk group, the top five genes were CTNNB1 (33%), TTN (25%), ALB (17%), TP53 (14%) and MUC16 (13%) (Fig. 5B). Notably, TP53, SPEG, TSC2, RB1and NLRP2 were more frequently mutated in the high-risk group compared to the low-risk group, whereas CDKN2A, CTNNB1, CACNA1B, and HECW2 exhibited higher mutation rates in the low-risk group (Fig. 5C-D). Although TMBs did not differ significantly between the two subgroups (*P* = 0.36) (Fig. 5E), patients in the high-risk group showed significantly elevated MATH scores compared to their low-risk counterparts (*P* = 0.0069), indicating increased intratumoral heterogeneity in the high-risk group (Fig. 5F).

### Prediction of therapeutic response

**Fig. 6:**
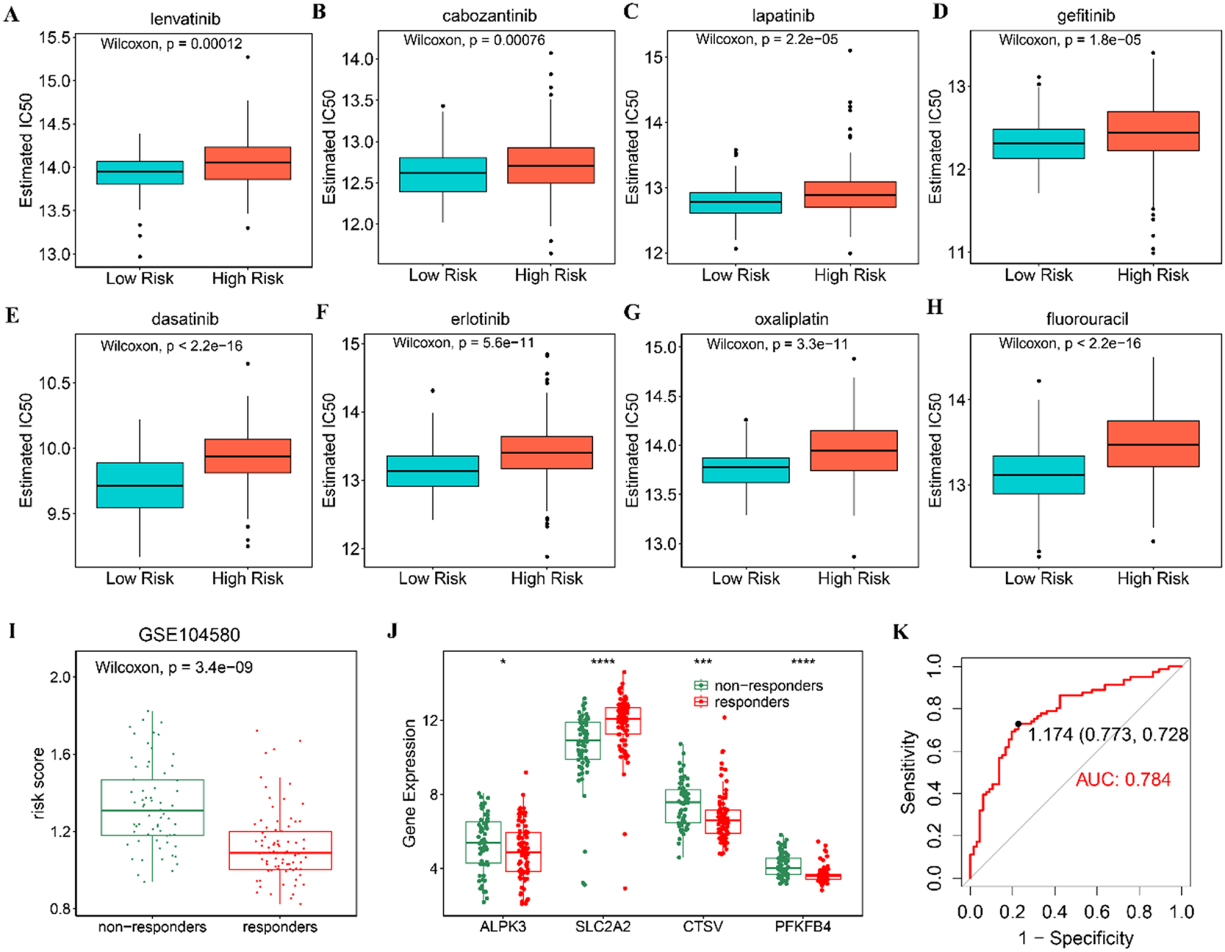
Treatment responses in high- and low-risk patients with HCC. **A**-H Chemotherapeutic and targeted therapeutic responses in high- and low-risk patients. **I** The risk scores for response and non-response groups. **J** The boxplot showing the expression of the selected 4 genes in responders and non-responders to TACE. **K** The AUC value of LRRPS in predicting efficiency of TACE in HCC patients. *P < 0.05, ***P < 0.001, **** *P* < 0.0001

We further investigated the association between risk scores and therapeutic response. Firstly, we analyze the therapeutic sensitivities of common chemotherapeutic and targeted drugs in HCC patients with different risk scores using the “oncoPredict” package. The findings indicated that patients in the low-risk group exhibited higher sensitivity to common targeted and chemotherapeutic medications for HCC, including lenvatinib, cabozantinib, lapatinib, gefitinib, dasatinib, erlotinib, fluorouracil, and oxaliplatin (Fig. 6A-H). Next, we assessed the correlation between LRRPS and the response to TACE treatment in the GSE104580 cohort, comprising 147 HCC patients who underwent TACE treatment. Notably, patients in the non-response group had significantly higher LRRPS (*P* < 0.001, Fig. 6I). Meanwhile, patients who did not respond to TACE exhibited higher expression levels of ALPK3, CTSV, and PFKFB4, while SLC2A2 showed increased expression in patients who responded to TACE treatment (Fig. 6J). The AUC value for predicting the TACE treatment response of HCC patients based on the LRRPS was 0.784 (Fig. 6K), further confirming the robust predictive capability of the risk score for therapeutic outcomes.

### Tumor microenvironment and stemness score analysis

**Fig. 7:**
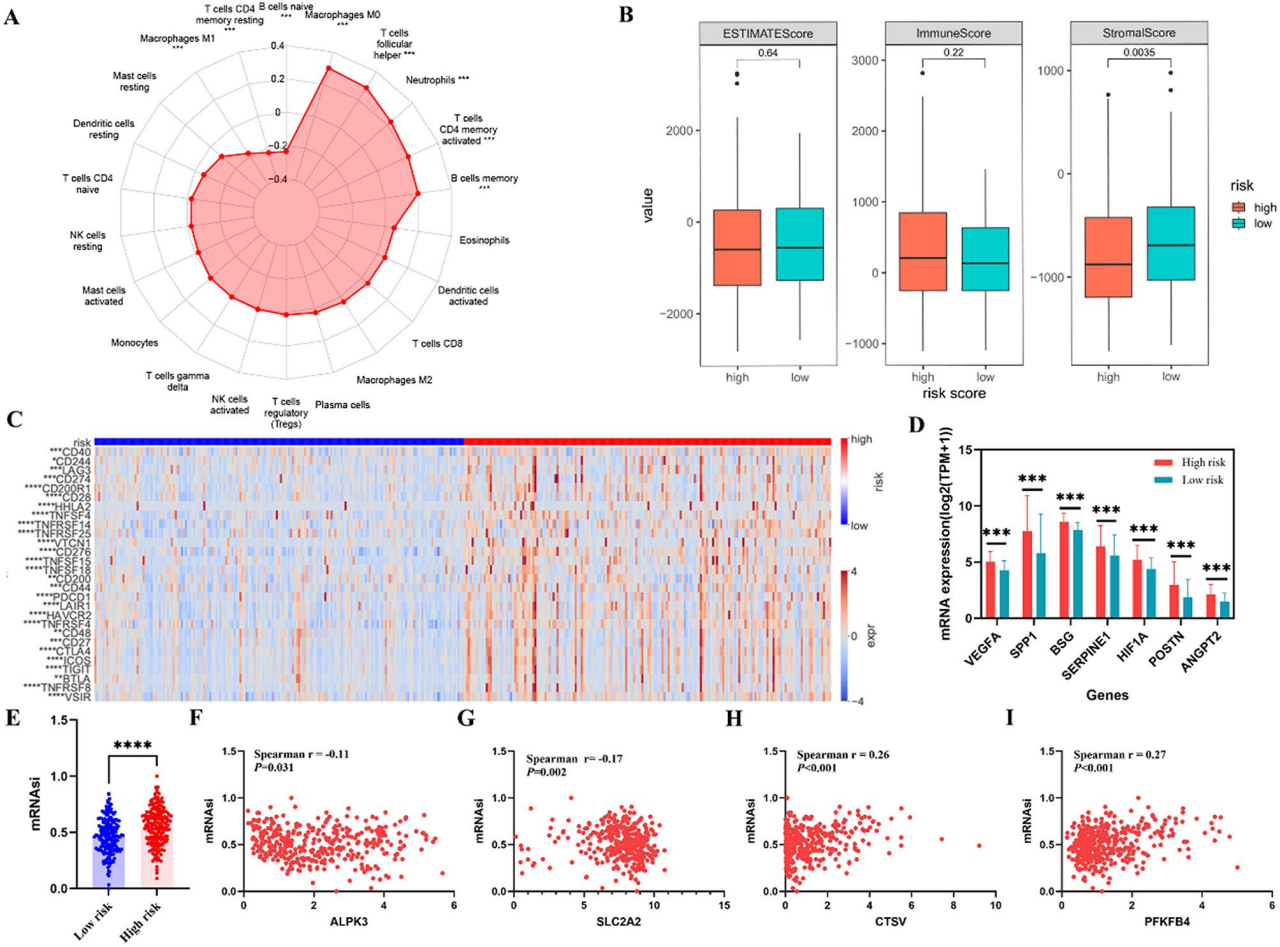
The tumor microenvironment and stemness score in high- and low-risk groups. **A** The correlation between the risk score and immune cell infiltration based on CIBERSORT analysis. **B** Comparisons of the ESTIMATE scores between the high- and low- risk groups. **C** Heatmap of immune checkpoints between high- and low-risk groups. **D** The association between risk score and angiogenesis-related genes. **E** The mRNAsi in different risk score groups. **F-I** The Spearman correlation analysis of mRNAsi with the expression of ALPK3(**F**), SLC2A2(**G**), CTSV(**H**) and PFKFB4(**I**). * *P* < 0.05, **P < 0.01, ***P < 0.001, **** *P* < 0.0001

The tumor microenvironment consists of the tumor’s surrounding components, including blood vessels, immune cells, fibroblasts, and the extracellular matrix, which are closely associated with tumor prognosis and treatment resistance [17]. Given the pivotal role of the risk score in prognostic prediction, we subsequently explored its potential in mirroring the tumor microenvironment. The CIBERSORT analysis unveiled a positive correlation between the risk score and the levels of M0 macrophages, T cells follicular helper, neutrophils, T cells CD4 memory activated, and B cell memory. Conversely, a negative association was observed with the infiltration of T cells CD4 memory resting, M1 macrophages, and B cells naïve (Fig. 7A). The findings implied that the infiltration of these immune cell subtypes may play a critical role in determining the prognosis of HCC patients. Moreover, the ESTIMATE analysis showed that patients classified as low-risk group exhibited elevated stromal scores compared to those in the high-risk group. However, there was no significant differences in either the ESTIMATE or immune scores between the two groups (Fig. 7B). In addition, we observed a positive correlation between expression levels of common immune checkpoint molecules and our risk score (Fig. 7C), suggesting that antitumor immunity may be impaired in high-risk patients. Blood vessels constitute an essential component of the tumor microenvironment, and angiogenesis playing a crucial role in tumor progression. Consequently, we examined the correlation between risk scores and well-established angiogenesis-related genes [18–20]. As shown in Fig. 7D, all of the angiogenesis-related genes were significantly overexpressed in the high-risk group. Cancer stem cells (CSCs) are recognized for their pivotal role in tumor initiation and their capacity to drive various malignant biological processes, including tumor recurrence and drug resistance. Additionally, cancer stemness is widely associated with detrimental effects on anticancer immunity and contributes substantially to the development of an immunosuppressive microenvironment [21, 22]. Therefore, we calculated the mRNAsi of HCC patients in TCCA database utilizing one-class logistic regression algorithm [14]. Our study revealed a significantly higher mRNAsi in high-risk patients compared to those in the low-risk group (Fig. 7E). Furthermore, we observed negative correlations between mRNAsi and the expression of both ALPK3 (Fig. 7F) and SLC2A2(Fig. 7G), while a significant positive correlation was found with the expression of CSTV (Fig. 7H) and PFKFB4 (Fig. 7I). Overall, these findings suggest that HCC patients in the high-risk group tend to exhibited a predominantly immunosuppressive tumor microenvironment.

### Functional enrichment analysis of HCC patients in high- and low-risk groups

**Fig. 8:**
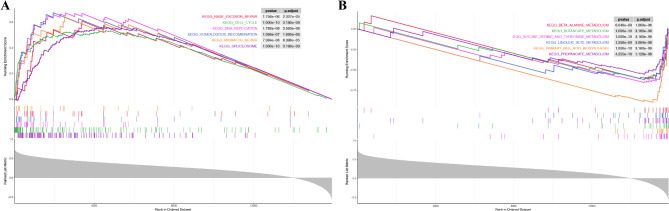
Results of the GSEA analyses. **A** The KEGG pathways activated in the high-risk group. **B** The KEGG pathway activated in the low-risk group

GSEA analysis revealed that activated pathways in the high-risk group were mainly associated with base excision repair, cell cycle, DNA replication, homologous recombination, mismatch repair, and spliceosome (Fig. 8A). While the pathways activated in the low-risk group involved more physiological functions of the liver (Fig. 8B).

### Internal and external validation of the values of these four genes in predicting prognosis

**Fig. 9:**
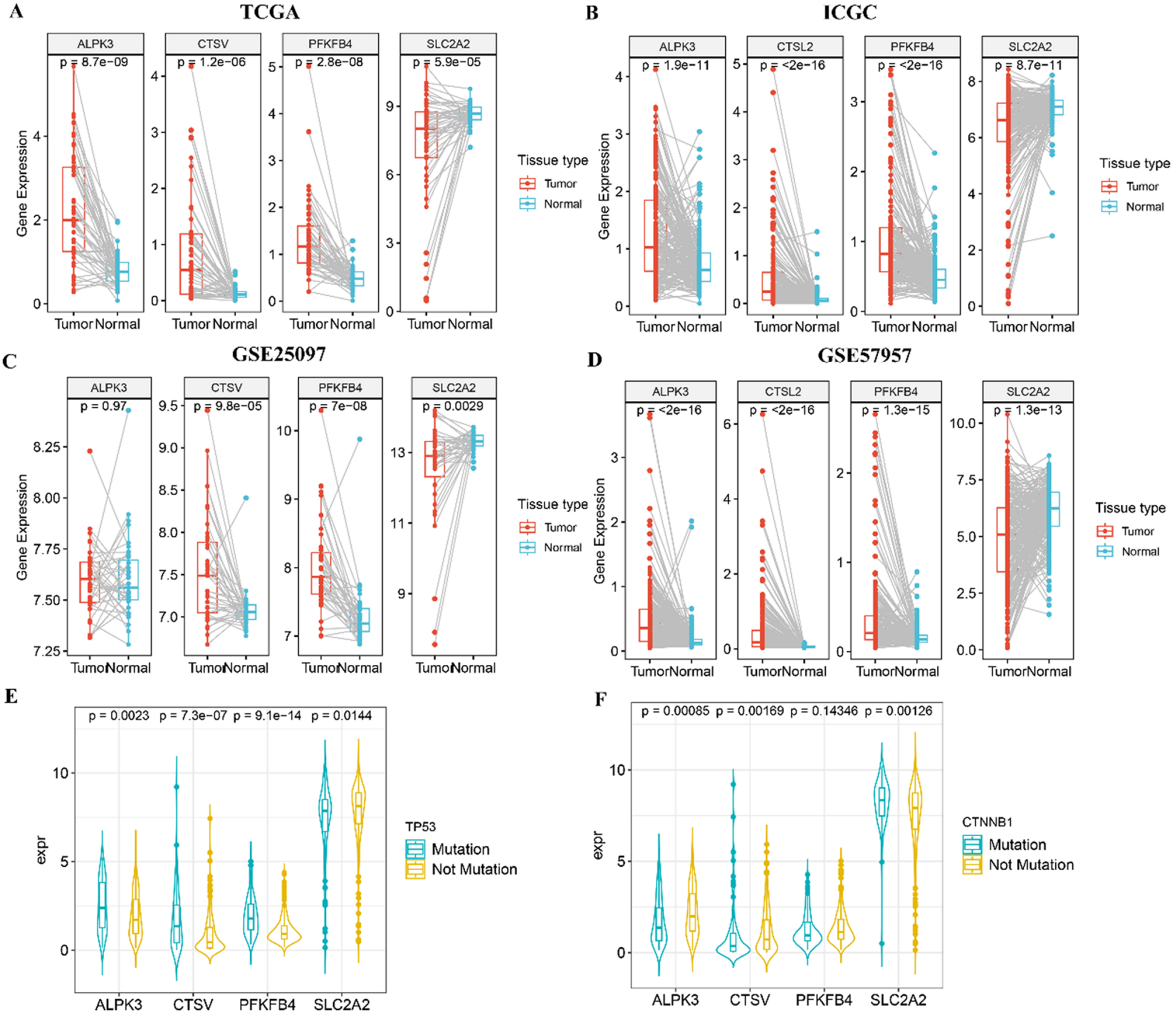
The expression and genetic alterations of the 4 selected genes. **A**-**D** Validations of the expression levels of the 4 selected genes in the TCGA-LIHC(**A**), ICGC(**B**), GSE25097(**C**) and GSE57957(**D**) datasets. **E**-**F** The association of selected 4 genes with TP53 mutation(**E**) and CTNNB1 mutation(**F**) in TCGA database

**Fig. 10:**
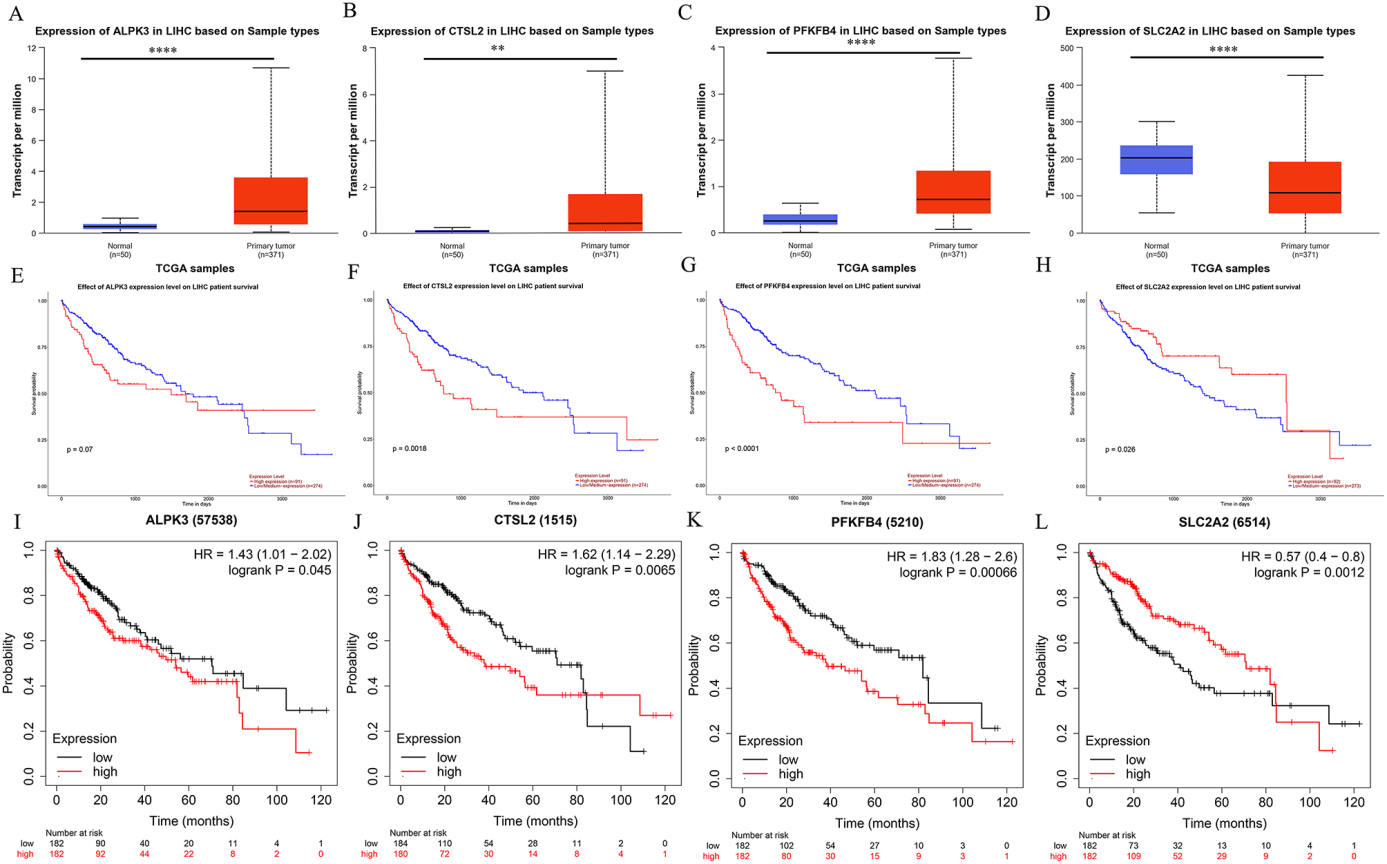
Validation of the expression and prognostic values of four genes in publicly available online databases. **A**-**D** Validation of the expression of the four genes in normal vs. tumor tissues in UALCAN database. **E**-**H** Validation of the value of the four genes in predicting the prognosis in UALCAN database. **I**-**L** Validation of the value of the four genes in predicting the prognosis in KM plotter database

To validate the robustness of the gene signature, we evaluated the expression and prognostic significance of the four genes across multiple datasets. Four datasets (TCGA, ICGC, GSE25097, and GSE57957) with matched tumor-normal samples were used to further assess the expression levels of these genes. The results showed that SLC2A2 was overexpressed in adjacent normal tissues, while the other three genes were upregulated in HCC tumor tissues (Fig. 9A-D). Additionally, these findings were validated using the ULCAN database (Fig. 10A-D, http://ualcan.path.uab.edu). Moreover, Kaplan-Meier analysis using the ULCAN database (Fig. 10E-H) and the KM plotter database (www.kmplot.com) (Fig. 10I-L) revealed that all genes, except for SLC2A2, were negatively correlated with patient OS. Our mutational analysis identified TP53 and CTNNB1 as the most frequently mutated genes in the high-risk and low-risk groups, respectively. Subsequently, we examined the expression profiles of the selected four genes under different mutation scenarios. As illustrated in Fig. 9E-F, ALPK3, CTSV, and PFKFB4 demonstrated significantly elevated expression in the TP53 mutant group, while showing markedly reduced expression in the CTNNB1 mutant group. Conversely, SLC2A2 exhibited significantly higher expression in the CTNNB1 mutant group, while demonstrating notably lower expression in the TP53 mutant group.

### PFKFB4 contributes to tumor progression and lenvatinib resistance in HCC

We selected PFKFB4, which exhibited the highest coefficient in the prognostic model, to further investigate its significance in predicting the prognosis of patients with HCC. Subsequent univariate and multivariate Cox regression analyses of TCGA database revealed that PFKFB4 (*P* < 0.001, HR = 1.455, 95%CI:1.207–1.753) serves as an independent predictor of overall survival in HCC patients (Table 1).

**Table 1:** Univariate and multivariate COX analyses of risk factors for OS in HCC patients

Parameters	Univariate Cox regression analysis			Multivariate Cox regression analysis		
	HR	95%CI	*P* value	HR	95%CI	*P* value
Age, years (≥ 60 vs. < 60)	1.214	0.856–1.722	0.278			
Gender (Male vs. Female)	0.816	0.573–1.163	0.260			
Tumor stage (III/IV vs. I/II)	2.449	1.689–3.549	< 0.001	2.000	1.354–2.955	**< 0.001**
Grade (G3/G4 vs. G1/G2)	1.120	0.781–1.606	0.539			
Vascular invasion (Y vs. N)	1.348	0.890–2.042	0.159			
AFP (ng/ml) (≥ 400 vs. < 400)	1.056	0.646–1.727	0.827			
PFKFB4 expression	1.566	1.334–1.840	< 0.001	1.455	1.207–1.753	**< 0.001**

**Fig. 11:**
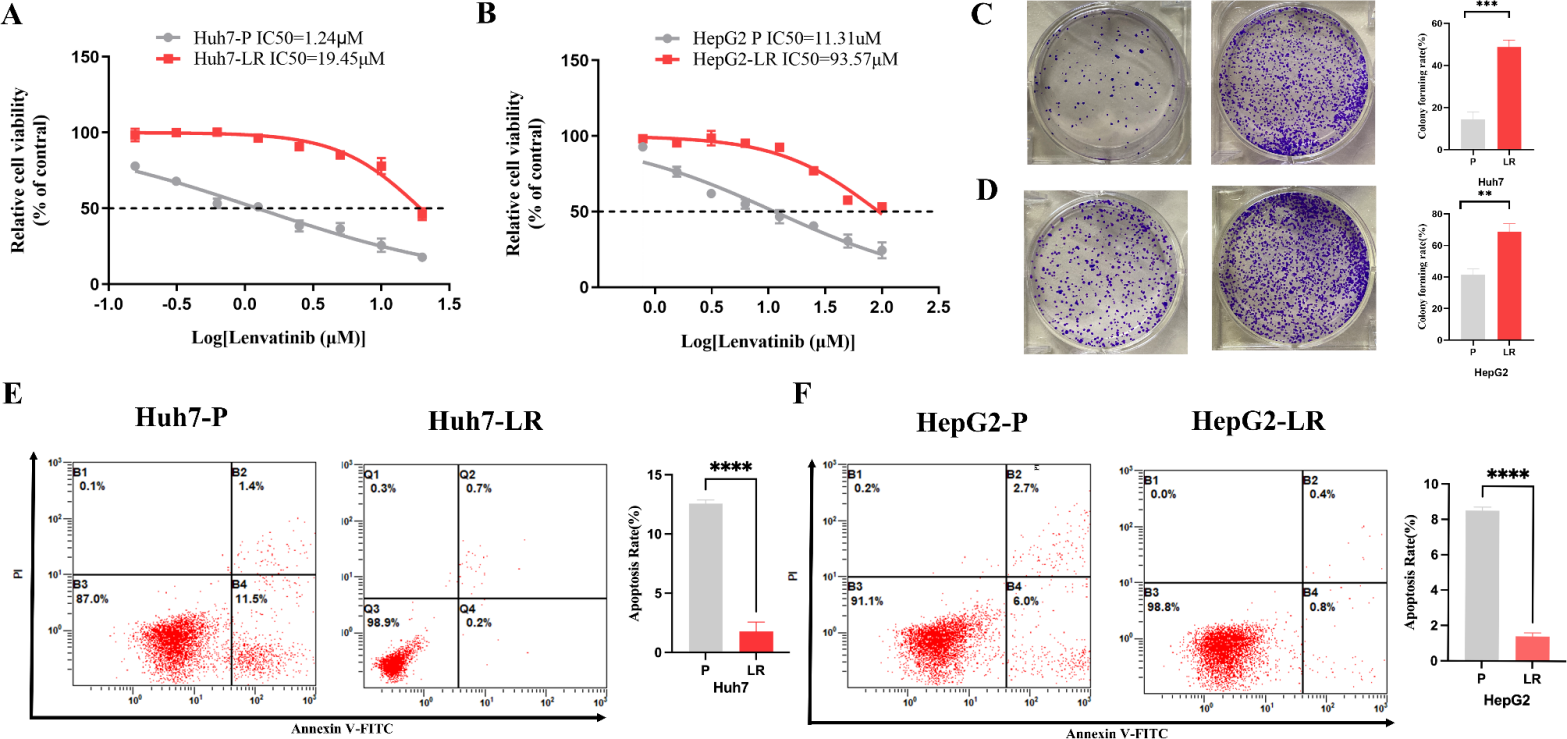
Successfully established LR cell lines. **A**, **B** The IC50 value of LR cells and their parental cells by CCK-8. **C**, **D** The colony forming ability of Huh7-LR and HepG2-LR cells and their parental cells under the treatment of 1µM and 10µM lenvatinib, respectively. **E**, **F** The apoptosis ability of Huh7-LR and HepG2-LR cells and their parental cells under the treatment of 1µM and 10µM lenvatinib, respectively. **P < 0.01, ***P < 0.001, **** *P* < 0.0001

**Fig. 12:**
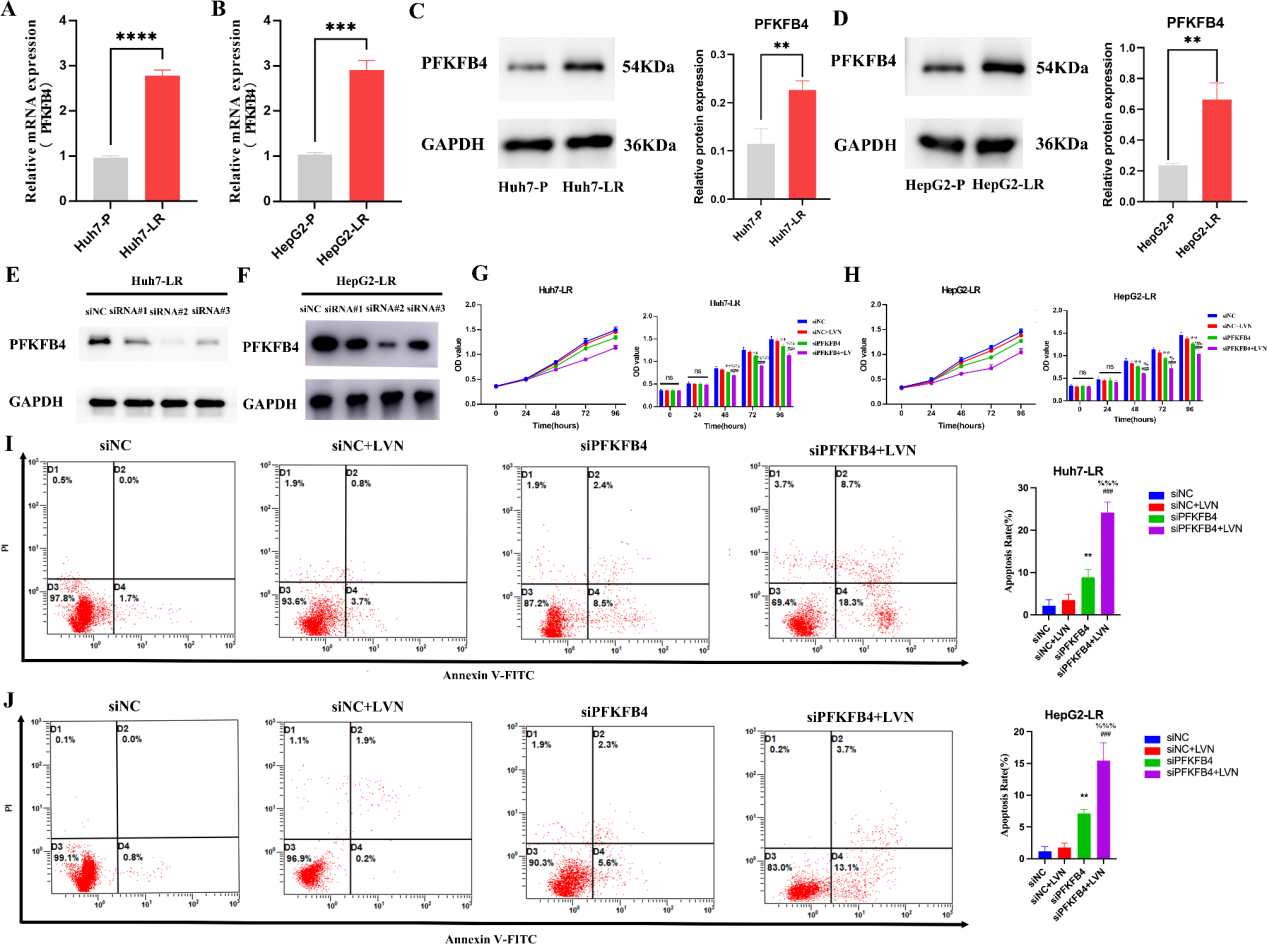
PFKFB4 contributes to lenvatinib resistance in HCC. **A**, **B** qRT-PCR showed the up-regulation of PFKFB4 in LR cells. **C**, **D** Western blot showed the up-regulation of PFKFB4 protein in LR cells. Western blot was performed using full-length and unprocessed membranes. **E**, **F** Western blot analysis of PFKFB4 knockdown efficiency of three PFKFB4-targeting siRNA in LR cells. Western blot analyses were performed using intact membranes for Huh7-LR cells(**E**), while stripped membranes was employed for experiments with HepG2-LR cells(**F**). **G**, **H** Cell viability was evaluated by CCK8 in different groups. **I**, **J** Cell apoptosis was detected by flow cytometry analysis in different groups. * siNC vs. siPFKFB4, # siNC vs. siPFKFB4 + LVN, % siPFKFB4 vs. siPFKFB4 + LVN, * *P* < 0.05, **P < 0.01, ***P < 0.001, **** *P* < 0.0001; # *P* < 0.05, ## *P* < 0.01, ### *P* < 0.001; % *P* < 0.05, %% *P* < 0.01, %%% *P* < 0.001

Given that PFKFB4 functions as an independent prognostic factor in HCC, we further investigated its role in lenvatinib resistance. We successfully established two lenvatinib-resistant cell lines by exposing the parental HCC cells to gradually increasing concentrations of lenvatinib, designated as Huh7-LR and HepG2-LR. The resistance of cells to lenvatinib was determined by CCK-8 assay. As shown in Fig. 11A and B, the IC50 values of lenvatinib for Huh7-LR and HepG2-LR cells were significantly higher than those of their parental cells. Lenvatinib-resistant cells exhibited a relatively higher colony forming ability (Fig. 11C, D) and anti-apoptotic potential than their parental cells (Fig. 11E, F) when exposed to equivalent concentrations of lenvatinib. These results suggested that the established LR strains could serve as a robust preclinical model for investigating the mechanisms underlying lenvatinib resistance. Thereafter, we used qRT-PCR and western blotting to evaluate PFKFB4 expression levels. The results showed significantly higher PFKFB4 mRNA levels (Fig. 12A, B) and protein levels (Fig. 12C, D) in Huh7-LR and HepG2-LR cells compared to their parental counterparts. To investigate whether PFKFB4 upregulation contributes to lenvatinib resistance, we utilized siRNA-mediated knockdown to reduce PFKFB4 expression in LR cells. Western blotting confirmed successful PFKFB4 knockdown in LR cells following siPFKFB4-2 transfection (Fig. 12E, F). PFKFB4 knockdown significantly inhibited the proliferation of LR HCC cells compared to the control group, whereas lenvatinib monotherapy exhibited no significant inhibitory effect. Notably, the combination of lenvatinib treatment and PFKFB4 knockdown exerted a synergistic antiproliferative effect on LR HCC cells (Fig. 12G, H). Consistently, lenvatinib alone failed to induce apoptosis in drug-resistant cells, while PFKFB4 knockdown significantly increased the apoptosis rate compared to the control group. Furthermore, the combination of lenvatinib and PFKFB4 silencing substantially enhanced apoptosis in drug-resistant cells (Fig. 12I, J). These findings indicate that PFKFB4 silencing enhances both the antiproliferative and pro-apoptotic effects of lenvatinib in LR cells. Collectively, our results identify PFKFB4 as a critical mediator of lenvatinib resistance in HCC and highlight its potential as a therapeutic target for overcoming lenvatinib resistance.

## Discussion

Hepatocellular carcinoma remains one of the most lethal malignancies worldwide, characterized by high morbidity and mortality rates [23]. The dismal prognosis of HCC is largely attributed to its aggressive biological behavior and marked resistance to conventional interventions. Lenvatinib has emerged as the standard first-line therapy for advanced HCC and has demonstrated notable efficacy. Nevertheless, a significant number of patients experience rapid development of drug resistance, leading to suboptimal outcomes [24]. It is well established that genes associated with drug resistance may also play a pivotal role in tumorigenesis and cancer progression. Previous studies have identified numerous gene signatures with predictive capabilities for the prognosis or therapeutic response of HCC [25–27]. However, the gene signature associated with lenvatinib resistance, serving as a predictor biomarker of prognosis and treatment efficacy in HCC, has been inadequately investigated. Therefore, our research focuses on developing a prognostic signature based on genes associated with lenvatinib resistance and exploring its predictive role in prognosis and treatment response, thereby facilitating the selection of optimal treatment and improving the clinical outcomes.

In this work, we have identified, for the first time, a lenvatinib resistance-related prognostic signature by integrating LRRDEGs with HCC prognosis-associated genes. Patients in the high-risk group exhibited advanced tumor staging and grading, elevated AFP levels, increased vascular invasion, and an unfavorable prognosis. Furthermore, high-risk patients tend to develop resistance to most molecular-targeted therapies and TACE treatments. Taken together, the new established 4-gene signature could not only predict the prognosis but also help select HCC patients with a high risk of drug resistance.

The nomograms constructed based on the risk score and TNM stage demonstrated enhanced prognostic predictive efficacy compared to conventional methods. While AFP is widely used as a diagnostic and prognostic biomarker for HCC, substantial controversy persists regarding the optimal cutoff value for predicting survival or recurrence. Furthermore, multiple studies have demonstrated that AFP alone possesses modest predictive power for HCC prognosis [28, 29]. In our study, the AFP level, with a cutoff value of 400 ng/ml, did not significantly impact the prognosis of patients with HCC in the TCGA cohort. Although TNM staging is the most commonly used system for prognostic prediction, our research demonstrates that both our nomogram and risk score model outperformed the conventional TNM staging system in predicting HCC prognosis. We acknowledge that validation of our predictive model in real-world clinical cohorts would further substantiate its practical utility. Consequently, our next step is to design a multicenter prospective study for the integration of the 4-mRNA panel with TNM staging for prognostic assessment in routine clinical practice. This prospective validation will further strengthen the clinical applicability and translational value of our model.

The identified prognostic signature holds significant potential for clinical decision-making. It may serve as a robust tool for stratifying patients and guiding clinicians in selecting optimal therapeutic strategies before treatment initiation. Specifically, patients with low-risk scores may respond well to conventional approaches, including lenvatinib, TACE or hepatic artery infusion chemotherapy with oxaliplatin and 5-fluorouracil. In contrast, high-risk patients, who are more likely to exhibit resistance to traditional treatments, may require alternative therapeutic strategies. Intriguingly, the risk score showed strong positive correlations with the expression of several immune checkpoint molecules, suggesting that high-risk patients may benefit from immune checkpoint inhibitors or combination immunotherapies (e.g., lenvatinib plus immune checkpoint inhibitors). Furthermore, since high-risk patients are more susceptible to developing drug resistance, clinicians should adopt more frequent monitoring protocols to detect resistance early and adjust treatment strategies accordingly. This personalized approach, guided by the lenvatinib resistance-related prognostic score, could improve therapeutic efficacy, reduce unnecessary adverse effects from ineffective treatments, and ultimately enhance clinical outcomes for HCC patients.

Given the excellent performance of this gene signature in predicting prognosis and treatment response in HCC, we explored its potential underlying mechanisms from following aspects: (1) The tumor microenvironment: The tumor microenvironment is composed of surrounding blood vessels, immune cells, stromal cells, signaling molecules, and extracellular matrix, which together drive tumor growth, metastasis, and drug resistance [30]. Our analysis revealed that the risk score positively correlated with infiltration levels of M0 macrophages (*r* = 0.30; *P* < 0.001), T cells follicular helper (*r* = 0.29; *P* < 0.001), neutrophils (*r* = 0.23; *P* < 0.001), T cells CD4 memory activated (*r* = 0.20; *P* < 0.001), and B cells memory (*r* = 0.20; *P* < 0.001). The majority of these cellular populations are protumor cells and contribute to immune escape. Pu et al. demonstrated that macrophage migration inhibitory factor is predominantly enriched in M0 macrophages, where it exerts a significant influence on tumor-associated immunosuppression and the intricate mechanisms of DNA repair [31]. Furthermore, a recent study found that M0 macrophages were significantly enriched in lung cancer patients with osimertinib resistance [32], suggesting that the immunosuppressive environment induced by macrophage infiltration is closely associated with therapeutic resistance. Increasing evidence supports neutrophils as key mediators of the immunosuppressive environment in which certain cancers develop, as well as drivers of tumor progression [33]. Moreover, Yi et al. revealed that lenvatinib-induced neutrophil extracellular traps (NETs) can inhibit cuproptosis in HCC cells, suggesting that targeting NETs may offer a promising strategy to overcome lenvatinib resistance in HCC [34]. Additionally, we observed that the risk score was negatively associating with M1 macrophages (*r*=-0.18; *P* < 0.001), T cells CD4 memory resting (*r*=-0.23; *P* < 0.001) and B cells naïve (*r*=-0.24; *P* < 0.001), indicating enhanced antitumor immune capacity in the low-risk group. Chen et al. found that siRNA-HIF-1α in combination with lenvatinib effectively inhibited tumor growth and prolonged the survival of tumor-bearing mice by increased the infiltration of T lymphocytes and M1 macrophages within the tumor microenvironment [35]. The above findings further indicate that these cells may attenuate tumor resistance and enhance therapeutic efficacy through their immunostimulatory effects. Notably, we found that multiple immune checkpoints, including PD-1(W = 11338; *P* < 0.001), CTLA-4(W = 10837.5; *P* < 0.001), LAG-3(W = 12932.5; *P* < 0.001), and TIGIT (W = 11551; *P* < 0.001), were significantly upregulated in the high-risk group. The high expression of immune checkpoint molecules enables tumor cells to evade immune surveillance, thus promoting tumor progression, which may partially explain the poor clinical outcomes observed in high-risk patients. Aberrantly proliferating blood vessels can restrict the entry of cytotoxic drugs and immune cells from the bloodstream into tumor tissue, thereby creating an immunosuppressive microenvironment and contributing to treatment resistance [36, 37]. Our analysis of the TCGA database revealed thatpatients with vascular invasion displayed significantly elevated LRRPS values compared to those without vascular invasion. Further investigation indicated a significant upregulation of angiogenesis-related genes in high-risk patients. Collectively, these findings suggest that high-risk HCC patients may exhibit an immunosuppressive tumor microenvironment, which could contribute to poor prognosis and therapeutic resistance. (2) Gene mutation: Genetic mutations critically influence tumor development and serve as valuable predictors of treatment response and prognosis. We observed a significantly higher TP53 mutation rate in high-risk patients compared to low-risk patients. This somatic mutation can lead to the inactivation of tumor suppressor genes and the induction of mutations in proto-oncogenes, thereby promoting tumorigenesis, inducing resistance, and contributing to poor prognosis [38, 39]. Intratumoral heterogeneity in HCC significantly impacts the tumor progression, metastasis, recurrence, and drug resistance [5]. MATH is a quantitative biomarker to assess intra-tumor heterogeneity. We observed significantly higher MATH scores in the high-risk group, further supporting its association with treatment resistance and poor prognosis. (3) Cancer stemness: Cancer stem cells are well-documented for their remarkable capacity to develop therapeutic resistance and poor outcomes [40]. Our study revealed a significantly higher mRNAsi in high-risk patients, reinforcing the utility of this risk score in predicting prognosis and treatment response. (4) DNA repair: DNA repair mechanisms are closely linked to drug resistance and reduced overall survival in HCC [41]. GSEA analysis revealed significant enrichment of two DNA repair-related pathways—base excision repair and mismatch repair—in high-risk patients, which may contribute to their increased susceptibility to drug resistance and poor prognosis. These comprehensive molecular analyses provide compelling evidence supporting the utility of this gene signature in predicting both prognostic outcomes and therapeutic responses in HCC patients.

The present prognostic model was established based on PFKFB4, CTSV, ALPK3, and SLC2A2. Our comprehensive analysis across multiple databases revealed that all genes, except for SLC2A2, were significantly upregulated in HCC tissues. Furthermore, we identified SLC2A2 as a protective factor for HCC, while the other three genes were associated with poor prognosis in HCC. Consistent with our findings, previous studies have demonstrated a significant upregulation of SLC2A2 in normal liver tissue compared to HCC tissue using immunohistochemistry [42]. Moreover, a pan-cancer analysis of SLC2A family genes found SLC2A2 were associated with the prolonged OS of HCC [43]. CTSV (also known as CTSL2), a member of the peptidase C1 family, encodes a lysosomal cysteine proteinase. CTSV was significantly upregulated in cancer tissue compared to adjacent normal tissue, with elevated CTSV expression correlating with adverse clinicopathological features and poor prognosis in HCC [44]. Recent research has further elucidated CTSV’s role in regulating cell cycle progression and proliferation, demonstrating its association with poor prognosis and tumor microenvironment modulation in HCC [45]. Additionally, He et al. demonstrated that CTSV knockdown synergistically enhanced the therapeutic efficacy of PD-1 inhibitors in HCC, suggesting a potential combinatorial approach for improving immunotherapeutic outcomes [46]. While ALPK3 has been extensively studied in hypertrophic cardiomyopathy [47, 48], its role in cancer remains largely unexplored. Lin et al. identified ALPK3 as a novel mutated gene in oral squamous cell carcinoma, suggesting its potential involvement in cancer development [49]. Our study revealed elevated ALPK3 expression in liver cancer tissues, correlating with poor prognosis in HCC patients. These findings warrant further investigation into the precise mechanisms linking ALPK3 to HCC pathogenesis.

PFKFB4, a member of the bisphosphatase enzyme family, functions as a pro-tumorigenic factor in various solid tumors [50, 51]. Previous studies have demonstrated PFKFB4 is markedly elevated in tumor tissue, correlating with aggressive tumor characteristics and adverse outcome [52, 53]. Consistent with these findings, our study identified PFKFB4 as an independent prognostic factor for poor survival in HCC patients. Notably, beyond its prognostic significance, our study explored the role of PFKFB4 in lenvatinib resistance in HCC. We provide novel evidence that PFKFB4 is upregulated in lenvatinib-resistant HCC cells and its silence effectively restores sensitivity to lenvatinib treatment. The most recent researches have revealed that activation of the HIF-1α pathway and glucose metabolism reprogramming play a crucial role in lenvatinib resistance in HCC [54, 55], both of which are intricately linked to PFKFB4. In future studies, we will establish a cell-derived xenograft tumor model using LR HCC cells transfected with shPFKFB4 to validate the contribution of PFKFB4 to lenvatinib resistance in vivo. Furthermore, we will conduct comprehensive analyses and rescue experiments to delineate the specific molecular mechanism involved in PFKFB4-mediated lenvatinib resistance in HCC. Particular attention will be given to PFKFB4-associated signaling pathways, including its interactions with HIF-1α, regulation of glucose metabolism reprogramming, and promotion of tumor stemness properties.

Despite conducting comprehensive analyses and validating our findings across multiple databases, several limitations in this study warrant acknowledgment. First, we constructed and validated the prognostic model using in silico analyses of public datasets, which may not fully represent the complexity and heterogeneity of human tumors. Patient heterogeneity, including genetic variability, differences in the tumor microenvironment, and diverse treatment histories, may influence the generalizability and predictive accuracy of our model across various clinical settings. We plan to design a multicenter prospective clinical trial to validate the robustness and clinical applicability of this model for predicting prognosis and guiding treatment decisions in routine clinical practice. Second, while our in vitro experiments demonstrate that PFKFB4 contributes to lenvatinib resistance, the precise underlying mechanisms require further elucidation through comprehensive in vivo and in vitro studies. Accumulating evidence suggests that PFKFB4 plays a crucial role in glycolytic metabolism, cancer stemness, and hypoxic adaptation [56–58], providing potential mechanistic insights into lenvatinib resistance. Future investigations will elucidate the specific molecular mechanisms by which PFKFB4 confers lenvatinib resistance in HCC, with particular emphasis on the aforementioned regulatory pathways.

## Conclusions

In conclusion, we established a robust prognostic model based on lenvatinib resistance-related genes, demonstrating its superior predictive value for patient prognosis and its potential to guide personalized treatment in HCC. Furthermore, PFKFB4 functions as a pivotal regulator in both lenvatinib resistance and HCC progression, highlighting its promise as a potential therapeutic target to overcome drug resistance and improve clinical outcomes in HCC patients.

## Electronic supplementary material

Below is the link to the electronic supplementary material.

Supplementary Material 1

## Data Availability

Data is provided within the manuscript or supplementary information files.

## Abbreviations

AFP: Alpha fetoprotein
AUC: Area under curve
CCK8: Cell counting kit-8
C-index: concordance index
DEGs: Differentially expressed genes
DMEM: Dulbecco’s modified Eagle’s medium
FDR: False discovery rate
GSEA: Gene set enrichment analysis
GEO: Genomics expression omnibus
HCC: Hepatocellular carcinoma
IC50: Inhibitory concentration
LIHC: Liver hepatocellular carcinoma
LR: Lenvatinib-resistant
LRRDEGs: Lenvatinib resistance-related differentially expressed genes
LRRPS: Lenvatinib resistance-related prognostic score
MATH: Mutant-allele tumor heterogeneity
mRNAsi: mRNA-based stemness index
OS: Overall survival
OD: Optical density
PCAs: Principal component analyses
ROC: Receptor operating characteristic
TACE: Transcatheter arterial chemoembolization
TCGA: The Cancer Genome Atlas
TGF-β: Transforming growth factor beta
TMB: Tumor mutation burden
TME: tumor microenvironment
TPM: Transcripts per kilobase million
TKIs: Tyrosine kinase inhibitors
