# Supplementary material for: Identification and validation of a lenvatinib resistance-related prognostic signature in HCC, in which PFKFB4 contributes to tumor progression and lenvatinib resistance
Source: BMC Gastroenterol. 2025 Apr 23;25:287. doi: 10.1186/s12876-025-03861-8 (PMC12020327; doi:10.1186/s12876-025-03861-8)
Supplement: Supplementary file 1 — Supplementary Material 1 [file 12876_2025_3861_MOESM1_ESM.docx]

Table S1. Summary of clinicopathologic data in TCGA and ICGC dataset.

|  |  | TCGA(n=365) | ICGC(n=231) |
| --- | --- | --- | --- |
| Age | >=60 | 200 | 187 |
|  | <60 | 165 | 44 |
| Gender | Male | 246 | 170 |
|  | Female | 119 | 61 |
| TNM Stage | Ⅰ+Ⅱ | 254 | 141 |
|  | Ⅲ+Ⅳ/Ⅲ | 87 | 90 |
|  | NA | 24 | 0 |
| Grade | G1 | 55 | NA |
|  | G2 | 175 | NA |
|  | G3 | 118 | NA |
|  | G4 | 12 | NA |
|  | NA | 5 | NA |
| Vascular invasion | Yes | 106 | NA |
|  | None | 54 | NA |
|  | NA | 205 | NA |
| AFP level | >=400ng/ml | 63 | NA |
|  | <400ng/ml | 213 | NA |
|  | NA | 89 | NA |
| Survival status | Dead | 235 | 42 |
|  | Alived | 130 | 189 |
